# Supplementary material for: Using child‐friendly movie stimuli to study the development of face, place, and object regions from age 3 to 12 years
Source: Hum Brain Mapp. 2022 Mar 11;43(9):2782–800. doi: 10.1002/hbm.25815 (PMC9120553; doi:10.1002/hbm.25815)
Supplement: Supplementary file 1 — Figure S1 Reverse correlation analysis of movie ssROIs for all age groups. For each plot, the x‐axis depicts the movie time in TRs (2 s per TR), and the y‐axis shows response magnitude. Line plots indicate the mean (z scored) time course of movie responses for each age group. Shaded blocks above time courses indicate timepoints marked as an “event” (i.e., a significant positive response across subjects; see Section 2) for each age group. [file HBM-43-2782-s001.docx]

**
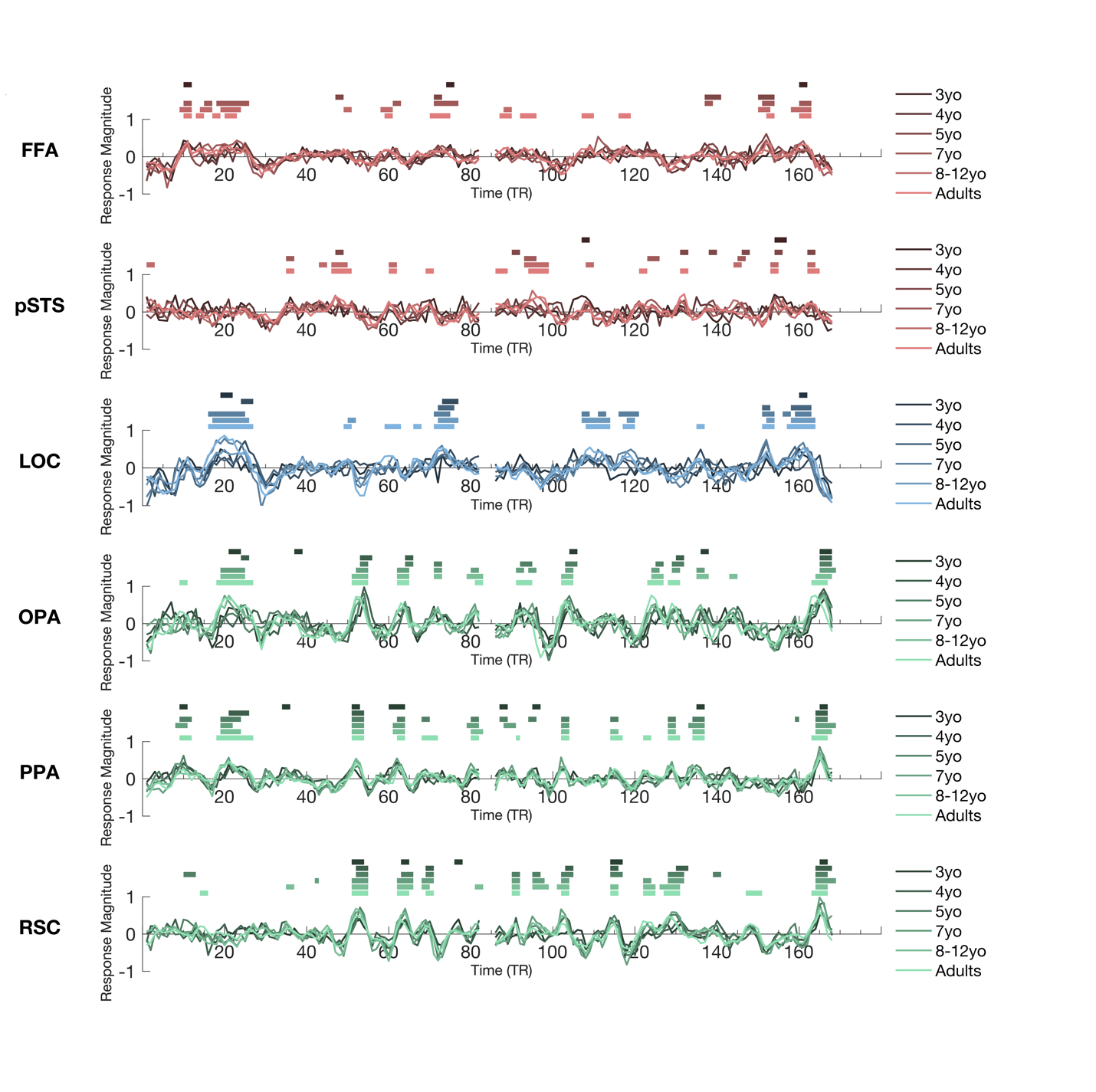
**

**Supplemental Figure 1. *Reverse correlation analysis of movie ssROIs for all age groups.***For each plot, the x-axis depicts the movie time in TRs (2s per TR), and the y-axis shows response magnitude. Line plots indicate the mean (z scored) time course of movie responses for each age group. Shaded blocks above time courses indicate timepoints marked as an “event” (i.e., a significant positive response across subjects; see Methods) for each age group.
